# Draft Genome Sequence of *Clostridium* sp. Maddingley, Isolated from Coal-Seam Gas Formation Water

**DOI:** 10.1128/genomeA.00081-12

**Published:** 2013-01-24

**Authors:** Carly P. Rosewarne, Paul Greenfield, Dongmei Li, Nai Tran-Dinh, Mark I. Bradbury, David J. Midgley, Philip Hendry

**Affiliations:** a CSIRO Animal, Food and Health Sciences, North Ryde, NSW, Australia; b CSIRO Mathematics, Informatics and Statistics, North Ryde, NSW, Australia

## Abstract

*Clostridium* sp. Maddingley was isolated as an axenic culture from a brown coal-seam formation water sample collected from Victoria, Australia. It lacks the solventogenesis genes found in closely related clostridial strains. Metabolic reconstructions suggest that volatile fatty acids are the main fermentation end products.

## GENOME ANNOUNCEMENT

A sample of coal-seam formation water was collected from Maddingley, Victoria, Australia (37°49′54″S, 144°25′23″E). Various physicochemical properties of the sample are described elsewhere (1). Under anoxic conditions the formation water was spread onto peptone-yeast extract-glucose (PYG) agar (1 g liter^-1^ peptone, yeast extract, and glucose plus 16 g agar liter^-1^) and incubated at 25°C until colonies appeared. One of the colonies propagated on the plates was purified by streaking and diluted to extinction 3 times in PYG broth. Genomic DNA was extracted using the Meta_G_Nome DNA isolation kit (Epicentre Biotechnologies).

The genome of *Clostridium* sp. Maddingley was sequenced using Illumina HiSeq. The resulting paired-end sequences were assembled using Velvet 1.1.07 into a draft genome containing 6,197,269 bp with a mean depth of coverage of ~1,400×. In total, the assembly comprised 184 large contigs (>200 bp) with a mean contig size of 33,514 bp, median of 11,634 bp, N50 of 88,157 bp, and a maximum length of 201,263 bp. The mean GC content of the genome was 29.8%. Annotation was performed using IMG ER (Integrated Microbial Genomes Expert Review) (2), which predicted a total of 5,778 protein-coding genes and 57 structural RNAs.

Based on comparison of the 16S rRNA (BLASTn) and HSP60 (BLASTx) genes (99% and 97% identity over 1,501 and 542 residues, respectively), *Clostridium* sp. Maddingley is closely related to *Clostridium beijerinckii* NCIMB 8502, a saccharolytic solventogenic bacterium that can produce a range of fermentation end products (3). Both genomes encode an extensive repertoire of carbohydrate active enzymes that are predicted to enable growth on substrates such as pentoses, hexoses, and starch. *Clostridium* sp. Maddingley also encodes several glycoside hydrolases (from families 5, 8, 10, 26, 28, 67, and 78) that are absent from the genome of *Clostridium beijerinckii* NCIMB 8502. It lacks the *sol* operon found in *C. beijerinckii* and other related species, including *C. acetobutylicum* and *C. saccharoperbutylacetonicum* (3–5), suggesting it is a nonsolventogenic isolate. Preliminary metabolic reconstructions indicate that volatile fatty acids are the main products of fermentation.

It is noteworthy that mixed-species anaerobic cultures enriched from the same formation water sample are capable of producing methane from a range of substrates, including brown coal, cellulose, and starch. Analysis of metagenomic data from the consortium suggests that *Clostridium* sp. Maddingley is the 4th most abundant taxon in the mixed-species enrichments (data not shown). We hypothesize that the products released by *Clostridium* sp. Maddingley during substrate fermentation may support growth of a hydrogenotrophic methanogen, *Methanobacterium* sp. Maddingley (1), in these enrichments. Such observations are central to understanding and improving the process of coal-seam methanogenesis *in situ*.

### Nucleotide sequence accession numbers.

This Whole Genome Shotgun project has been deposited at DDBJ/EMBL/GenBank under the accession no. ALXI00000000. The version described in this paper is the first version, ALXI01000000.
